# To the Memory of H. M. Golding, D.F.C., of the Royal Flying Corps

**Published:** 1971-01

**Authors:** 


### To the memory of H. M. Golding, D.F.C., of The Royal Flying Corps

### The Dawn Patrol

Once you dared to fly on wings made
Of frail fabric stretched on frames of wood;
White wings that dizzily dipped and swayed,
As you soared alone on the dawn patrol;
Far below you saw the dawn-mists rise,
To shield the shell-pocked earth and shattered trees
From the sad gaze of the comprehending skies—
Grim monuments to war's black-death disease:
Fokker fighters dived in fierce attack,
And flaming tracer bullets pierced your wings;
You flash-turned, fired, and sent them reeling back,
And not till your task was done did you turn from the flack—
To fly, a bird that guns had failed to destroy,
Winging your way o'er the woodlands shadowy green,
Rising and falling in the gusts in utter joy,
And away o'er the flower-strewn fields to the drome that was home:
Peace, and yours the vigil of the true physician,
With each humble home your temple of healing, touching—
As divine music the mind of a deaf musician—
The impalpable chords of the immaterial world:
Now you've flung aside the chrysalis of night,
And questing beyond the stars your distant goal,
You've raised your gallant wings in eager flight,
And on that dawn patrol you are not alone.

